# SV2A expression in blood cells as a possible biomarker candidate for levetiracetam treatment response

**DOI:** 10.1002/epi.70122

**Published:** 2026-02-04

**Authors:** Johannes Lang, Mathias Linnerbauer, Jeanne Cuny, Veit Rothhammer, Hajo Hamer

**Affiliations:** ^1^ Epilepsy Center, Department of Neurology, University Hospital Erlangen Friedrich Alexander University Erlangen–Nuremberg Erlangen Germany; ^2^ Deutsches Zentrum Immuntherapie Uniklinikum Erlangen Erlangen Germany

**Keywords:** drug efficacy, levetiracetam, peripheral blood mononuclear cells, synaptic vesicle membrane protein 2A, human, treatment response

## Abstract

**Objective:**

This study was undertaken to evaluate whether synaptic vesicle protein 2A (SV2A) expression on peripheral immune cells predicts treatment response to levetiracetam in epilepsy.

**Methods:**

High‐dimensional flow cytometry was used to prospectively assess SV2A expression on immune cells from levetiracetam responders, nonresponders, and healthy controls. SV2A expression levels were further validated using real‐time quantitative polymerase chain reaction (RT‐qPCR) in an independent retrospective cohort. The predictive value of SV2A expression on naive CD8^+^ T cell‐specific SV2A/CD3^+^ median fluorescence intensity (MFI) ratio was determined, and correlations with central nervous system (CNS)‐resident cell expression were examined in the wild‐type (WT) C57BL6 mouse model.

**Results:**

Naive CD8^+^ T cells showed significantly lower SV2A expression (*p* = .0029) in nonresponders compared to responders, confirmed by RT‐qPCR (*p* = .0022), with no difference in overall CD8^+^ T‐cell abundance. The naive CD8^+^ T cell‐specific SV2A/CD3^+^ MFI ratio (>.4733) correctly identified most responders, with a positive predictive value of 81.8%. SV2A concentration was stable over time (mean interval = 121.2 days, 95% confidence interval = 93.64–148.7 days), unaffected by age, gender, dose, or treatment duration, and neuronal SV2A expression in the CNS of WT C57BL6 mice correlated with SV2A expression of CD8^+^ in circulating blood cells (*r* = .655, *p* = .008).

**Significance:**

Naive CD8+ T cell‐specific SV2A MFI ratio may represent a potential indicator of treatment response for levetiracetam. Pending further validation, its stability and accessibility suggest that it could potentially support treatment decisions and help to reduce ineffective drug trials.

**Key points**

- The expression of SV2A in peripheral blood mononuclear cells correlates significantly with the protein's expression in brain tissue.
- The expression in naive CD8+ T cells is significantly reduced in patients responding poorly to levetiracetam (<50% reduction of seizures).
- Cell‐specific SV2A MFI discriminates between responders and nonresponders (AUC = .77).
- SV2A expression levels in peripheral blood may help predict levetiracetam treatment response (PPV > 80%).

## 1 INTRODUCTION

Epilepsy is a prevalent chronic neurological disorder that affects approximately 68 million people of all ages worldwide.^1^ The mainstay of treatment is symptomatic and based on the pharmacological suppression of epileptic seizures.^2^ Substances suppressing seizure activity are collectively referred to as antiseizure medications (ASMs), and they have been demonstrated to be an efficacious treatment, with the potential to achieve long‐term remission (i.e., seizure freedom) in approximately 60%–70% of all patients.^2^, ^3^ In recent years, newer ASMs with favorable pharmacokinetics, improved tolerability, and reduced drug interactions have emerged and have become incorporated into the daily clinical routine, some of which addressed new therapeutic targets with distinct mechanisms of action. Levetiracetam (LEV) is a ligand to the synaptic vesicle protein 2A (SV2A),^4^, ^5^ a vesicle transmembrane protein that is expressed in all synapses of the central nervous system (CNS) and plays a priming role in neural vesicle fusion.^6^ SV2A regulates the quantity of fusion‐competent vesicles and is believed to modulate the formation of soluble N‐ethylmaleimide sensitive factor attachment receptor complexes that bridge opposed lipid layers during vesicle fusion.^7^, ^8^ Albeit LEV failed to demonstrate noninferiority for time to 12‐month remission at the group level in patients with newly diagnosed focal epilepsy and was significantly more likely to fail due to adverse reactions than first‐line therapy with lamotrigine, it is widely prescribed due to its high tolerability, efficacy, and ease of use.^9^ Nevertheless, to the best of our knowledge, there is no test currently available that can predict the potential efficacy of any ASM, or LEV in particular, in individual people with epilepsy (PWE) before treatment initiation. Therefore, apart from recommendations on the choice of ASM for epilepsy types and syndromes at a population level, individual patients and their caregivers are frequently compelled to engage in a process of trial and error when selecting any ASM over the course of their disease. In an attempt to investigate a potential immunomodulatory effect of LEV as a cause of the increased incidence of upper respiratory tract infections, Li et al.^10^ described SV2A expression in CD8^+^ T cells and observed a decreased degranulation of CD8^+^ T cells following LEV treatment. The objective of this study was to demonstrate that the level of SV2A expression on peripheral CD8^+^ T cells is a suitable biomarker for predicting the response of epileptic seizure frequency to LEV.

## 2 MATERIALS AND METHODS

### 2.1 Patient cohorts and collection

People with epilepsy were included following admission to the epilepsy center or presentation in the outpatient clinic at the Department of Neurology at University Hospital Erlangen. Patients who were taking LEV at the time of admission were included in a retrospective group, whereas patients who were about to start treatment were included into a prospective study group. The clinical decision for the introduction of or a medication switch to LEV was made independent of the study, and all patients gave informed written consent before inclusion. Patients were categorized as responders (RESs) and nonresponders (NRs) based on their response to treatment. A patient was considered an RES if he or she showed a greater than 50% reduction in seizure frequency while taking the medication and a 50%–75% reduction of seizure frequency was recorded after at least 12 months compared to the time of inclusion. Patients were considered seizure‐free if they had no clinical history of seizures (100% reduction) for ≥5 consecutive years. In addition, suspected healthy control (HC) individuals without any known medical condition were included into an HC group. Whole blood samples (9 mL) were taken at the time of inclusion (retrospective group) or at every consultation (prospective group) from patients and controls.

### 2.2 Mice

Mice were housed as previously described.^11^ In brief, two to five animals per cage under a standard light cycle (12:12 h light:dark; lights on from 07:00 a.m. to 7:00 p.m.) at 20–23°C and humidity of ~50% were provided with ad libitum access to water and food. Adult female mice 8–12 weeks old were used on a C57Bl/6J background (The Jackson Laboratory, #000664).

### 2.3 PluriBead cell isolation

Human whole blood peripheral blood mononuclear cells (PBMCs) were separated according to clusters of differentiation (CD) through positive selection using nonmagnetic antibody‐coated plastic beads (PluriSelect) and cell strainers. Separation using CD4+, CD8+, CD2+, and CD14+ was carried out according to the manufacturer's protocol. After selection, cells were stored at –80°C for later processing.

### 2.4 Isolation of circulating cells from mouse

Whole blood was collected from the apex of the left ventricle prior to transcardiac perfusion and collected in EDTA‐coated 1.5‐mL Eppendorf tubes. Red blood cells were lysed with ACK lysing buffer (Life Technology, A10492‐01) for 5 min, washed with 1× phosphate‐buffered saline (PBS), and prepared for downstream applications.

### 2.5 Isolation of splenic cells from mouse

Splenic cells have been isolated as previously described.^11^ Spleens were mechanically dissected and dissociated by passing through a 100‐μmol·L^−1^ cell strainer (Thermo Fisher Scientific, 10282631). Red blood cells were lysed with ACK lysing buffer (Life Technology, A10492‐01) for 5 min, washed with 1× PBS, and prepared for downstream applications.

### 2.6 Isolation of CNS cells from mouse

Cells from the CNS of mice were isolated as previously described.^11^ Mice were perfused with cold 1× PBS, and the CNS was isolated and mechanically diced using sterile razors. Brains and spinal cords were processed separately or pooled (if not indicated otherwise) and transferred into 5 mL of enzyme digestion solution consisting of 35.5 μL papain suspension (Worthington, #LS003126) diluted in enzyme stock solution (ESS) and equilibrated to 37°C. ESS consisted of 10 mL 10× Earle balanced salt solution (EBSS; Sigma‐Aldrich, #E7510), 2.4 mL 30% D(+)‐glucose (Sigma‐Aldrich, #G8769), 5.2 mL 1 mol·L^−1^ NaHCO3 (VWR, #AAJ62495‐AP), 200 μL 500 mmol·L^−1^ EDTA (Thermo Fisher Scientific, #15575020), and 168.2 mL ddH2O, filter‐sterilized through a .22‐μm filter. Samples were shaken at 80 rpm for 30–40 min at 37°C. Enzymatic digestion was stopped with 1 mL of 10× high ovomucoid inhibitor solution and 20 μL .4% DNase (Worthington, #LS002007) diluted in 10 mL inhibitor stock solution (ISS); 10× high ovomucoid inhibitor stock solution contained 300 mg bovine serum albumin (Sigma‐Aldrich, #A8806) and 300 mg ovomucoid trypsin inhibitor (Worthington, #LS003086) diluted in 10 mL 1× PBS and filter sterilized using a .22‐μm filter. ISS contained 50 mL 10× EBSS (Sigma‐Aldrich, #E7510), 6 mL 30% D(+)‐glucose (Sigma‐Aldrich, #G8769), and 13 mL 1 mol·L^−1^ NaHCO3 (VWR, #AAJ62495‐AP) diluted in 170.4 mL ddH2O and filter sterilized through a .22‐μm filter. Tissue was mechanically dissociated using a 5‐mL serological pipette and filtered through a 70‐μm cell strainer (Thermo Fisher Scientific, #22363548) into a fresh 50‐mL conical tube. Tissue was centrifuged at 600 × *g* for 5 min and resuspended in 10 mL of 30% Percoll solution (9 mL Percoll [GE Healthcare Biosciences, #17–5445‐01], 3 mL 10× PBS, 18 mL ddH2O). Percoll suspension was centrifuged at 600 × *g* for 25 min with no breaks. Supernatant was discarded, and the cell pellet was washed once with 1× PBS, centrifuged at 500 × *g* for 5 min, and prepared for downstream applications.

### 2.7 RNA isolation and real‐time quantitative polymerase chain reaction

RNA isolation and real‐time quantitative polymerase chain reaction (RT‐qPCR) was performed as previously described.^11^ In brief, cells were lysed in 350 μL Buffer RLT (Qiagen), and RNA was isolated using the RNeasy Mini Kit (Qiagen, #74004) according to the manufacturer's instructions. Five hundred nanograms of RNA from each sample was transcribed into cDNA using the High‐Capacity cDNA Reverse Transcription Kit (Life Technologies, #4368813). Gene expression was assessed by qPCR using the TaqMan Fast Advanced Master Mix (Life Technologies, #4444556). The following TaqMan probes were used: SV2A human Hs01059458_m1 (Thermo Fisher Scientific, #4331182), GAPDH human Hs02786624_g1 (Thermo Fisher Scientific, #4331182), ACTB human Hs01060665_g1 (Thermo Fisher Scientific, #4331182).

### 2.8 Flow cytometry

Flow cytometry was performed as previously described.^11^ Antibodies used in this study are listed in detail in the supplementary material.

For intracellular flow cytometry staining, cells were fixed over night after surface staining using the eBioscience Foxp3/Transcription Factor Staining Buffer Set (eBioscience, #00552300) according to the manufacturer's instructions. For staining of intracellular cytokines, the following antibodies were used: PE‐eFlour610‐iNOS (eBioscience, #61592080, 1:100), BV711‐IL17a (BioLegend, #506941, 1:100), AF488‐HB‐EGF (Santa Cruz Biotechnology, #sc‐365182 AF488, 1:100), FITC‐CXCL12 (Thermo Fisher Scientific, #MA523547, 1:100), PE‐Cy5‐FoxP3 (Thermo Fisher Scientific, #15–5773‐82, 1:200), PE‐Cy7‐IFNγ (BioLegend, #505826, 1:100), PE PerCP‐eFlour710‐TNF (eBioscience, #46732180, 1:200), APC‐GM‐CSF (eBioscience, #17733182, 1:100), and APC‐eF780‐Ki67 (Thermo Fisher Scientific, #506941, 1:100).

### 2.9 Analysis of multiparameter flow cytometry data

Data were analyzed using the OMIQ platform as previously described.^11^ For dimensionality reduction, cells were downsampled to an appropriate number per group. Uniform Manifold Approximation and Projection (UMAP) was performed using the following parameters: 15 neighbors, minimum distance = .4, 200 epochs.

To investigate the cell types with the most significant differences in SV2A expression between RESs, NRs, and HCs, we performed multiclass Significance Analysis of Microarray (SAM)^12^ based on the median fluorescence intensity (MFI). SAM 19 was performed on groups using a multiclass approach (with maximum 100 permutations and a false discovery rate cutoff of .1).

### 2.10 Standard protocol approvals, registrations, and patient consent

The collection and study of human blood samples was approved by the ethics committee of the University of Erlangen under the registration number 95_12 B. Experiments on human tissue were performed in accordance with the Declaration of Helsinki. The animal study was approved by Bavarian State Authorities (Regierung von Unterfranken, TS‐09/2020 Neurologie).

### 2.11 Statistical analysis

For the statistical analysis of two groups, a two‐sample unpaired *t*‐test was performed. Familywise significance and confidence level was set at *p* < .05. If not otherwise described, statistical examinations were carried out using GraphPad Prism 9 (v.9.5.1). Linear regression analysis was performed using GraphPad Prism 9 (v.9.5.1). Additional information on the study design and the statistical tests used are provided in the figure legends. Graphs and illustrations were created using Adobe Illustrator (v.1.0.).

## 3 RESULTS

### 3.1 Patient cohort and clinical characteristics

We sampled 164 individuals who visited our Epilepsy Center between February 2015 and July 2017 and between May 2021 and November 2023. Six patients had to be excluded due to insufficient data to determine their responder status (*n* = 4), for being seizure‐free without medication during follow‐up (*n* = 1), and because the diagnosis of epilepsy turned out to be incorrect and was subsequently classified as an acute symptomatic seizure (*n* = 1). The final cohort consisted of 158 individuals, a total of 121 PWE and 37 HCs (Table 1). Of these, 102 were recruited for flow cytometry‐based analysis (53 male, 49 female, mean age = 37.4 years, range = 18–86, 49 RESs, 31 NRs, and 22 HCs), and 56 were recruited for polymerase chain reaction‐based analysis (30 male, 26 female, mean age = 39.0 years, range = 18–70, 23 RESs, 18 NRs, 15 HCs).

**TABLE 1:** Clinical characteristics of study cohort.

Characteristic	All	People with epilepsy		Healthy controls
		Responders	Nonresponders	
Cohort, *n*	158	72	49	37
Age, years, mean (range)	38.0 (18–86)	38.9 (18–80)	38.5 (18–86)	35.4 (18–58)
Gender, F/M	77/94	28/44	24/25	23/14
Epilepsy onset, years (range)	28.8 (1–86)	29.4 (1–80)	27.9 (4–86)	
Epilepsy duration, years (range)	10.0 (0^a^–54)	9.5 (0^a^–54)	10.7 (0^b^−46)	
Epilepsy syndrome, Foc/MFoc/Gen/U	93/1/26/2	54/0/17/1	39/1/9/1	
Hemisphere involved [L/R/B/U]	41/26/6^c^/22	27/13/1/14	14/13/5^c^/8	
ADR, *n* (%)	37 (31%^d^)	20 (28%^d^)	17 (35%^d^)	

Abbreviations: ADR, adverse drug reaction; B, bilateral; F, female; Foc, focal; Gen, generalized; L, left; M, male; MFoc, multifocal; R, right; U, unclassified.

^a^ .0027 = 1/365, 1 day.

^b^ .019 = 7/365, 7 days.

^c^ One patient suffered from hypothalamic hamartoma with seizure propagation to the frontobasal cortex on both hemispheres.

^d^ Percent of 121 people with epilepsy, 72 responders, and 49 nonresponders, respectively.

**FIGURE 1:**
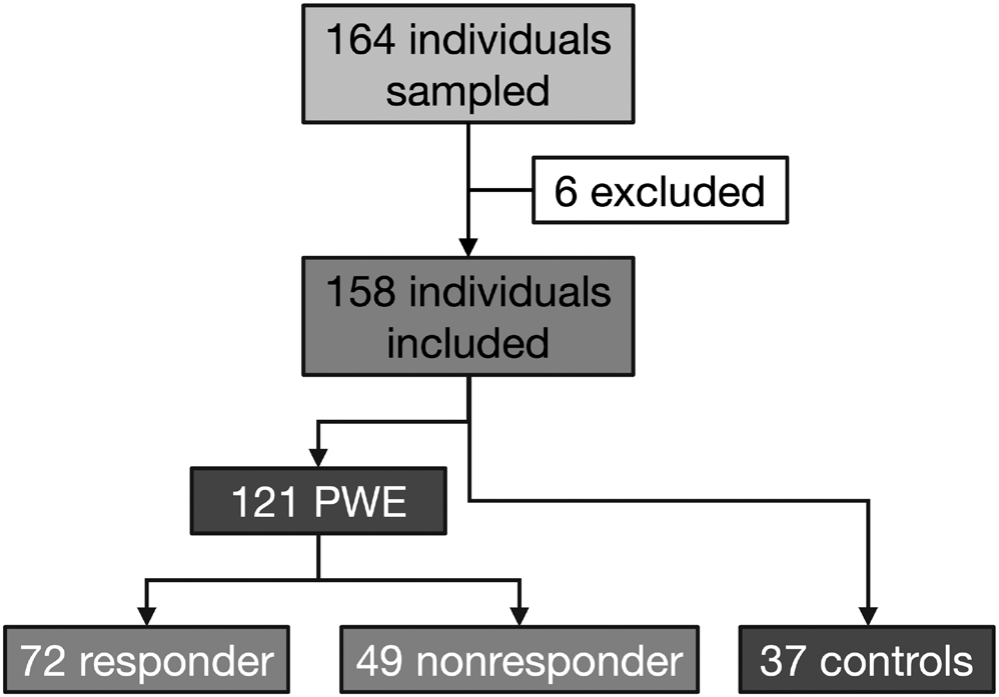
Study cohort. A total of 164 individuals were sampled, of whom 158 were included in the final study cohort for further analysis. One hundred twenty‐one were people with epilepsy (PWE), of whom 72 responded under levetiracetam with reduction of seizure frequency > 50% (responders), and 49 did not (<50%, nonresponders).

Regarding type of epilepsy, 92 PWE were diagnosed with focal epilepsy, of whom 24 suffered from unclear etiology. One patient was multifocal due to periventricular nodular heterotopias (PVNH6, mutation in the *ERMARD* gene), 26 PWE had generalized epilepsy, and two remained unclassified (Table 1). Among the PWE with generalized epilepsy, four were diagnosed with juvenile myoclonic epilepsy and one was diagnosed with Jeavons syndrome with absence seizures and eyelid myoclonia. Over the course of the study, 32 (of the 158) participants (six HCs) were sampled at least twice, and of these eight (one HC) were sampled three times. Seventy‐two PWE (60%) responded to LEV (>50% reduction of seizure frequency), and 49 (40%) did not (Table 1). Among the patients who responded to LEV treatment, 45 (37%) achieved complete seizure freedom (Figure 1).

Thirty‐seven PWE (31%) experienced CNS adverse drug reactions (ADRs), of whom 20 were among RESs (28%) and 17 were among NRs (35%), without a significant difference between the two groups (*p* = .3910), in type of epilepsy (focal vs. generalized, *p* = .7292), or in LEV dosage (*p* = .6645). In addition, we found no difference in naive CD8+ T cell‐specific SV2A MFI ratio between PWE with and without CNS ADRs (*p* = .6180; Figure S1A–D). More than 50% of patients experiencing CNS ADRs suffered from depression (*n* = 20, 54%), of whom four had suicidal thoughts (LEV mean dosage = 2440 mg, range = 750–4000), followed by increased aggression (*n* = 19, 51%), fatigue (*n* = 14, 38%), impaired cognition (*n* = 11, 30%), and other CNS ADRs (*n* = 5, 14%).

### 3.2 High‐dimensional immunophenotyping in levetiracetam RESs versus NRs

**FIGURE 2:**
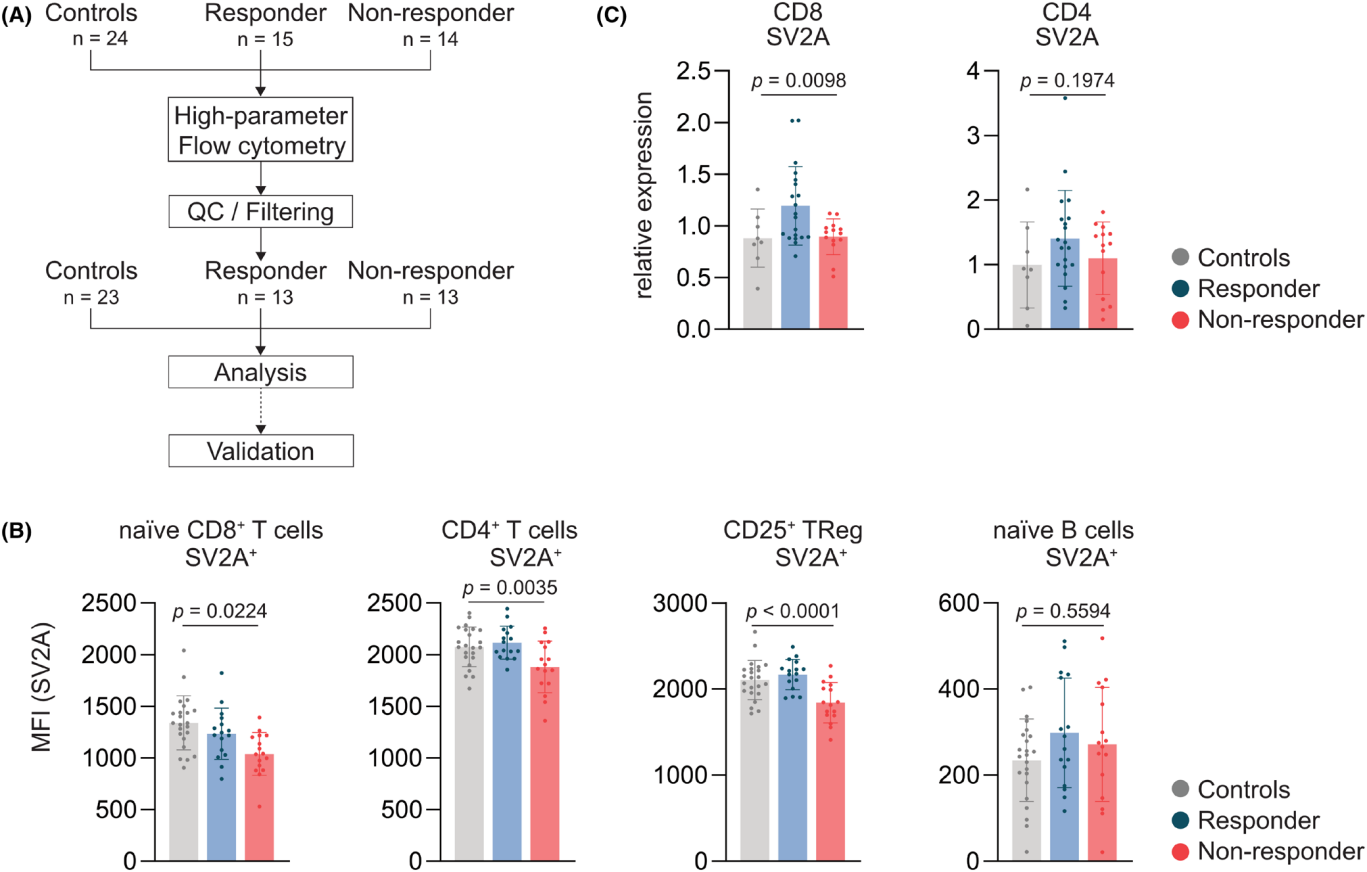
Phenotyping of immune cells in levetiracetam (LEV) responder and nonresponder patients. (A) Blood immunophenotyping by high‐dimensional flow cytometry was performed in 24 control patients, 15 LEV responder people with epilepsy (PWE), and 14 nonresponder PWE. Following filtering and quality control (QC), levels of synaptic vesicle protein 2a (SV2A) were analyzed. (B) Median fluorescence intensity (MFI) of SV2A expression by naive CD8^+^ T cells, CD4^+^ T cells, CD25^+^ T_Reg_ cells, and naive B cells in controls (*n* = 23), responder patients (*n* = 13), and nonresponder patients (*n* = 13). Unpaired *t*‐test was used to compare responders and nonresponders. (C) Real‐time quantitative polymerase chain reaction analysis of SV2A expression by CD8^+^ CD4^+^ sorted cells from controls (*n* = 8), responder patients (*n* = 20), and nonresponder patients (*n* = 14). Unpaired *t*‐test was used to compare between responders and nonresponders.

Using high‐dimensional flow cytometry of circulating cells, we investigated differences in immune cell composition in people with epilepsy with different responses to LEV in 14 NRs, 15 RESs, and 24 control patients. Following filtering and quality control, 13 NRs, 13 RESs, and 23 HCs remained in the cohort for further analyses (Figure 2A). Whereas no differences in immune cell abundance could be observed between HCs and RESs (.4444, 95% confidence interval [CI] = −1.970 to 2.859, *p* = .9022, nonsignificant), NRs demonstrated a reduction in CD4^+^ T cells when compared to HCs (3.886, 95% CI = .9629–6.809, *p* = .0053) and RESs (−3.442, 95% CI = −6.598 to .2853, *p* = .0286; Figure S2A,B). UMAP of SV2A MFI demonstrated high expression of the protein by neutrophils and T cells (Figure S2C,D).

Among SV2A‐expressing cell types, naive CD8^+^ T cells, naive CD4^+^ T cells, overall CD4^+^ T cells, and naive B cells revealed the largest differences in SV2A expression between LEV RESs, LEV NRs, and HCs (Figure S2E). This was confirmed by manual gating, demonstrating a significant decrease in SV2A expression by naive CD8^+^ T cells (CD8^+^ CD45RA^+^ CCR7^+^ CD27^+^), and CD4^+^ T cells of NRs compared to RESs, whereas the expression of SV2A by naive B cells (CD19^+^ CD27^−^ IgD^+^) remained unchanged between the groups (Figure 2B and Figure S2F). Notably, among CD4^+^ T cells, particularly CD25^+^ regulatory T (T_Reg_) cells displayed reduced levels of SV2A expression in NRs (Figure 2B). These differences in SV2A expression by naive CD8^+^ T cells, and CD25^+^ T_Reg_ cells were independent of gender, CNS side effects, age, duration of LEV treatment, and LEV dosage (Figure S3A,B).

To validate the different expression of SV2A on the mRNA level in an independent retrospective cohort, we isolated CD4^+^ and CD8^+^ T cells of eight HCs, 20 RESs, and 14 NRs, and quantified the expression of SV2A by RT‐qPCR; CD8^+^ T cells isolated from NR patients demonstrated a reduced expression of SV2A compared to RESs, whereas no differences were observed in isolated CD4^+^ T cells (Figure 2C).

### 3.3 CD8‐specific SV2A as predictor of LEV response

**FIGURE 3:**
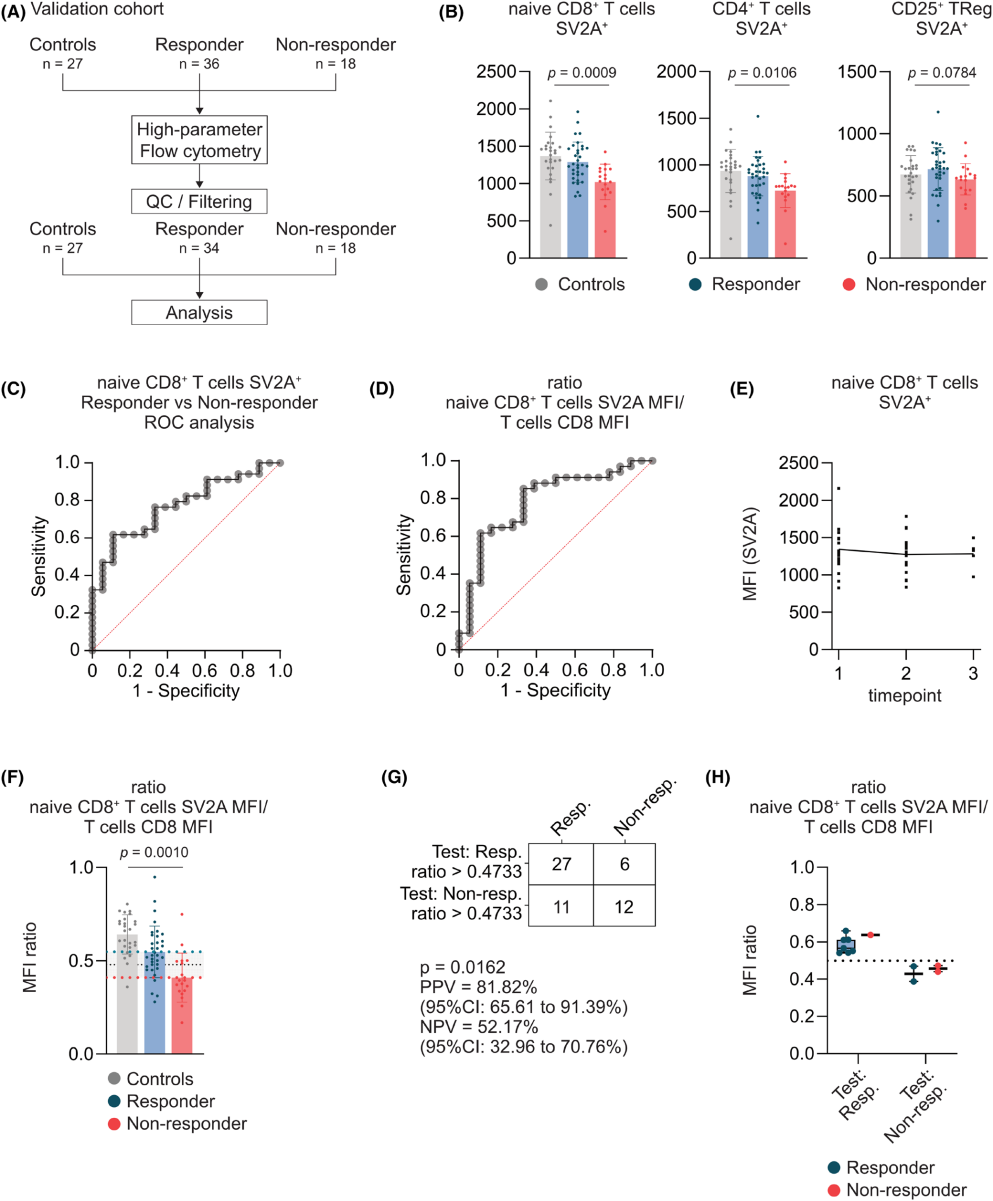
Validation and prediction of treatment response. (A) Flowchart of validation cohort. Blood immunophenotyping by high‐dimensional flow cytometry was performed in 27 control patients, 36 levetiracetam responder people with epilepsy (PWE), and 18 levetiracetam nonresponder PWE. Following filtering and quality control (QC), levels of synaptic vesicle protein 2a (SV2A) were analyzed. (B) Median fluorescence intensity (MFI) of SV2A expression by Naive CD8^+^ T cells, CD4^+^ T cells, and CD25^+^ T_Reg_ cells in controls (*n* = 27), responder patients (*n* = 34), and nonresponder patients (*n* = 18). Unpaired *t*‐test between responders and nonresponders. (C) Receiver operating characteristic (ROC) curve describing SV2A expression (MFI) by naive CD8^+^ T cells as classifier for levetiracetam responders versus nonresponders. (D) ROC curve describing ratios between naive CD8^+^ T‐cell SV2A MFI and CD3^+^ T‐cell CD8 MFI as classifier for levetiracetam responders versus nonresponders. (E) Longitudinal assessment of naive CD8^+^ T‐cell SV2A MFI levels in 18 patients with at least two sampling timepoints. (F) Ratio between naive CD8^+^ T‐cell SV2A MFI and CD3^+^ T‐cell CD8 MFI in controls (*n* = 27), responder patients (*n* = 34), and nonresponder patients (*n* = 18). Unpaired *t*‐test was used to compare responders and nonresponders. (G) Contingency test for predicted versus observed response to levetiracetam. Predicted response was defined as naive CD8^+^ T‐cell SV2A MFI/CD3^+^ T‐cell CD8 MFI ratio above or below .0100. Fisher exact test (two‐sided) was applied. (H) Predicted versus observed response in 12 prospective patients with previously unknown response to levetiracetam based on a naive CD8^+^ T‐cell SV2A MFI/CD3^+^ T‐cell CD8 MFI ratio above or below .4733. CI, confidence interval; Non‐resp., nonresponders; NPV, negative predictive value; PPV, positive predictive value; Resp., responders.

To establish a threshold of SV2A expression that can be used for the prediction of treatment response to LEV, we performed high‐dimensional flow cytometry on circulating immune cells of 27 HCs, 34 RESs, and 18 NRs (Figure 3A). Because the frequency of SV2A^+^‐naive CD8^+^ T cells in LEV NRs was significantly decreased compared to LEV RESs (Figure S4A), we decided to use MFI for further analyses, based on its capacity to obviate the necessity for manual gating of a positive population while robustly reflecting SV2A expression levels. In line with our previous observations, we found a significant decrease of SV2A expression by SV2A MFI in naive CD8^+^ T cells and CD4^+^ T cells of LEV NRs in comparison to LEV RESs, but not CD25+ T_Reg_ cells (*p* = .0009, *p* = .0106, and *p* = .0784, respectively; Figure 3B).

Due to the consistency in CD8+ cells, we sought to assess whether naive CD8^+^‐specific SV2A MFI levels are of prognostic relevance to determine treatment response to LEV and found that naive CD8^+^‐specific SV2A MFI levels effectively discriminated between LEV RES and NR patients, with an area under the curve (AUC) of .77 (*p* = .0048; Figure 3C, Figure S4B). Similarly, naive CD8^+^‐specific SV2A MFI levels obtained from the analysis of a confirmational cohort achieved a prognostic prediction, with an AUC of .89 (*p* = .0007; Figure S4C,D).

Because MFI values can vary between cytometers and experiments, we next normalized naive CD8^+^ T‐cell SV2A MFI levels to CD3^+^ T‐cell CD8 MFI levels, a measurement that remained stable between groups (Figure S4E). Similar to absolute MFI values, the ratio between naive CD8^+^ T‐cell SV2A MFI levels and CD3^+^ T‐cell MFI levels achieved a prognostic prediction, with an AUC of .78 (*p* = .0005; Figure 3D, Figure S4F).

To investigate whether naive CD8^+^‐specific SV2A MFI levels remain stable over time and in response to treatment, we assessed the longitudinal change of naive CD8^+^‐specific SV2A MFI levels in 18 patients who were sampled multiple times, nine (50%) of whom were therapy naive at their first sampling timepoint (mean time between sampling timepoints = 121.2 days, 95% CI = 93.64–148.7 days). Naive CD8^+^ T‐cell SV2A levels remained stable over time (Figure 3E, Figure S4G).

Next, we defined a cutoff value for the MFI ratio of .4733 based on the group means of LEV RES (mean = .5379, 95% CI = .4862–.5895) and LEV NR (mean = .4087, 95% CI = .3189–.4733) patients (Figure 3F), allowing us to test the predictive capability of naive CD8^+^‐specific SV2A MFI levels for all previously measured patient samples, where an MFI ratio < .4733 would suggest a limited response to LEV, and an MFI ratio > .4733 would indicate a reduction of seizure frequency > 50%. Contingency analysis revealed a positive predictive value (PPV) of 82% (95% CI = 65.61–91.39) and negative predictive value (NPV) of 52% (95% CI = 32.96–70.76, *p* = .0100; Figure 3G, Figure S3H).

**FIGURE 4:**
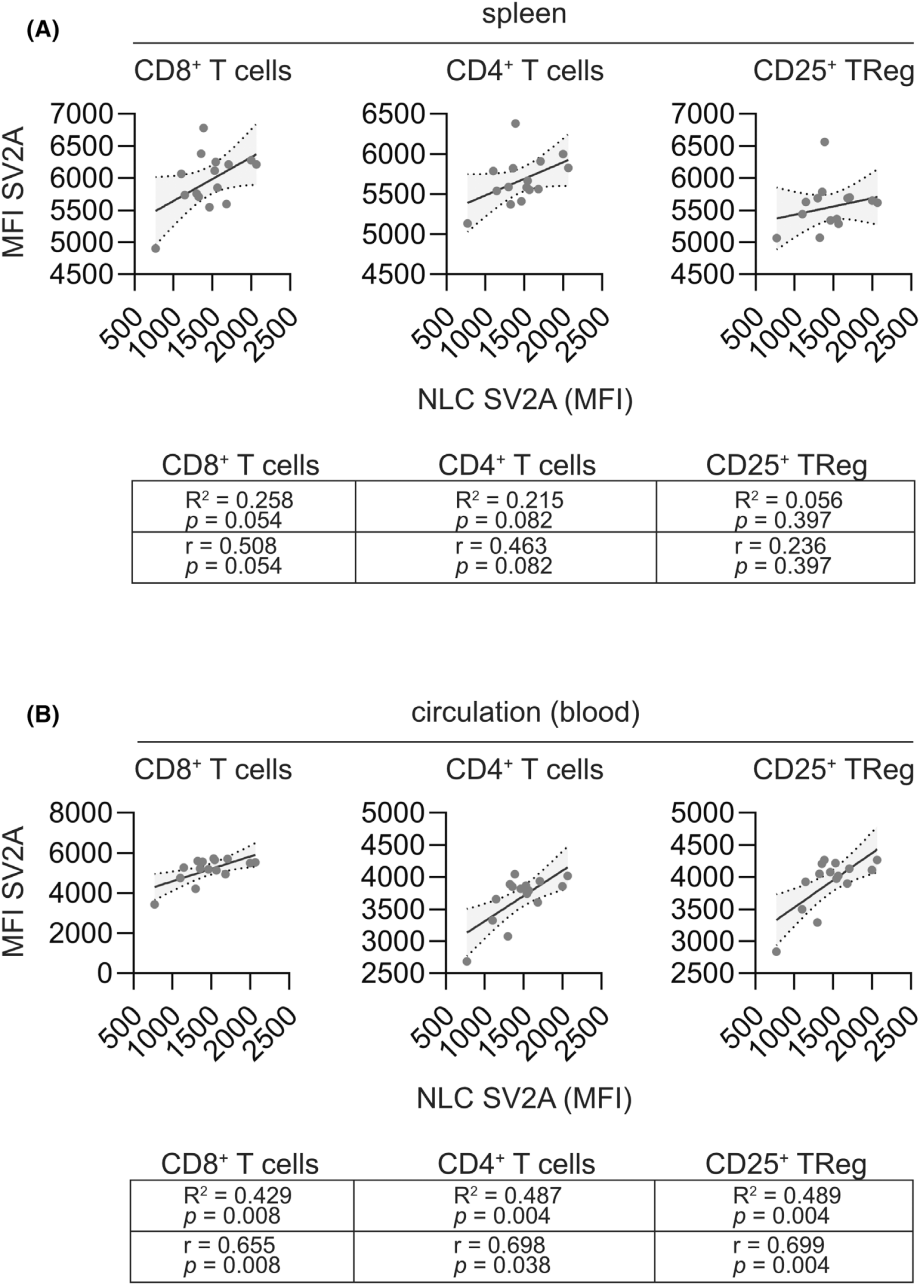
Correlation between peripheral and central nervous system‐specific synaptic vesicle protein 2A (SV2A) expression. (A) Linear regression and Pearson analysis of SV2A expression (median fluorescence intensity [MFI]) in splenic CD8^+^ T cells, CD4^+^ T cells, and CD25^+^ T_Reg_ cells in respect to SV2A expression (MFI) by neuronal lineage cells (NLCs) in wild‐type (WT) mice. *n* = 15. (B) Linear regression and Pearson analysis of SV2A expression (MFI) in circulating (blood) CD8^+^ T cells, CD4^+^ T cells, and CD25^+^ T_Reg_ cells in respect to SV2A expression (MFI) by NLCs in WT mice. *n* = 15.

Finally, we assessed the MFI ratio in a subgroup of 16 therapy‐naive patients stratified according to their treatment response to LEV during follow‐up. In these patients, MFI ratio revealed a PPV of 71.43% (95% CI = 35.89–94.92) and an NPV of 62.50% (95% CI = 30.57–86.32, *p* = .3147; Figure 4H). In this smaller yet purely prospective subgroup, two thirds were still correctly identified as RESs with a lower sensitivity, but there was a higher specificity in identifying PWE with poor response to LEV, which might be more relevant when avoiding unnecessary drug trials is prioritized (data not shown).

### 3.4 SV2A expression in periphery and CNS correlate

Increased SV2A expression in glutamatergic synaptic terminals was described as a potential determinator of response to LEV in a Wistar rat model of temporal lobe epilepsy.^13^ To evaluate whether the expression of SV2A by peripheral immune cells correlates with the expression of SV2A by CNS‐resident cell types and thus can serve as a predictive biomarker for LEV treatment response in epilepsy, we performed flow cytometric analyses of tissue‐resident splenic immune cells, circulating immune cells, and CNS‐resident cells in wild‐type (WT) C57BL6 mice (Figure S5A–C). In line with prior observations, neuronal lineage cells (NLCs) demonstrated the highest expression of SV2A, followed by astrocytes, oligodendrocyte lineage cells, and endothelial cells (Figure S5B). Linear regression and Pearson correlation analysis revealed no significant correlation between the expression of SV2A by NLCs and tissue‐resident CD8^+^ T cells, CD4^+^ T cells, or CD25^+^ T_Reg_ cells in the spleens of WT mice (Figure 4A). However, we observed a significant correlation between SV2A expression by NLCs and the expression of SV2A by circulating CD8^+^ T cells, CD4^+^ T cells, or CD25^+^ T_Reg_ cells (Figure 4B).

## 4 DISCUSSION

In this study, we aimed to identify a prognostic biomarker that can be used to robustly determine response to LEV early on during treatment to improve clinical decision‐making. Although an effect of LEV on CD8^+^ cell degranulation was shown a decade ago,^10^ this has not been investigated for use as a biomarker of treatment efficacy. Using high‐dimensional immunophenotyping, we defined the expression of the synaptic protein SV2A on peripheral immune cells as a predictive biomarker for LEV treatment response in patients with epilepsy.

The potential relevance of our findings is underlined in that despite major international efforts to improve the treatment of epilepsy in recent decades, improving seizure control is offset by a reduced quality of life and shortened life expectancy of up to 10 years in PWE with symptomatic epilepsy and 2 years for idiopathic or cryptogenic epilepsy.^14^ This is reflected by a higher standardized mortality ratio compared to the general population.^15^ In 2000, Kwan and Brodie published a single‐center study of 525 patients with newly diagnosed epilepsy treated with ASM, of whom 63% remained seizure‐free during follow‐up.^16^ More recently, after a further 14 years, they followed up a larger cohort of 1795 patients, showing that despite the introduction of newer ASM over the past 3 decades, a similar proportion of patients achieved seizure freedom with the first ASM.^17^ For the remaining approximately one third, the likelihood of achieving seizure control with each additional ASM decreased to 12% and 4% for the second and third drugs, respectively. In addition, although a collective of >80% achieved seizure freedom with the first to third ASM, only 50% remained seizure‐free for 1 year or longer, highlighting that many PWE struggle to find effective long‐term treatment. Thus, it would be of great clinical value if an appropriate treatment strategy could be determined other than in a probatory manner to improve outcomes and minimize adverse effects and factors associated with prolonged treatment. Attempts to train support vector machines with clinical and electrographic features to predict treatment response to LEV achieved prediction accuracy of 90%, primarily based on features of the frontopolar beta band.^18^ However, this study was based exclusively on retrospective data and did not control for the same features in healthy subjects.

Expression profiling of SV2A on more than 20 immune cell populations in RESs, NRs, and HCs in our study revealed that both CD4^+^ and CD8^+^ lymphocytes are capable of expressing the synaptic protein, representing the first large‐scale analysis of SV2A expression by PBMCs in humans. Although abundance of CD8^+^ T cells did not differ between the groups, CD4^+^ T cells showed a significant reduction in NRs compared to RESs and HCs using high‐dimensional flow cytometry. Furthermore, we validated these findings by isolating CD8^+^ and CD4^+^ T cells in an independent retrospective cohort by examining the SV2A expression using RT‐qPCR, which clearly showed a reduced expression of SV2A in CD8^+^ T cells isolated from NRs compared to RESs, but not in isolated CD4^+^ T cells. These findings make CD4^+^ T cells seem less suited for comparing expression levels between these groups. The concordance between the SV2A expression levels of CD8^+^ T cells, CD4^+^ T cells, and CD25^+^ T_Reg_ cells in RESs and HCs and the comparatively lower expression in PWE experiencing treatment failures (NRs), as well as the interindividual variation of the expression levels, suggest that exceeding a threshold value relative to CD3+ cells (MFI ratio) could be a suitable indicator of a good treatment response, which in our study was >.4733. Based on the SV2A MFI ratio in CD8^+^ T cells, we could identify the majority of LEV‐responding PWE, with a PPV of 82%, warranting the use of the naive CD8^+^ T‐cell SV2A MFI/CD3^+^ T‐cell CD8 MFI ratio as a predictive marker for response to LEV. For an ASM such as LEV, which in recent years has emerged as an effective treatment for several types of epilepsy and has thus become a widely used drug, a reduction in the number of applications for NRs may represent a clinically significant improvement, so that many PWE potentially eligible for LEV could have received another ASM early on, sparing them a frustrating drug trial.

In contrast to other ASMs, LEV acts by binding to SV2A, preventing calcium release from intraneuronal stores, and thereby counteracting excessive synchronized neuronal activity. As previously described, LEV alters the activation and effector functions of CD8^+^ T cells,^10^ potentially through SV2A‐dependent inhibition of calcium.^19^ Previous reports have suggested that SV2A levels in CNS glutamatergic terminals correlate with the response to LEV,^13^ but these levels are difficult and impractical to measure. Conversely, our objective was to establish a peripheral proxy for CNS‐specific SV2A levels that is easy to access (from peripheral blood) and thus could be used in clinical routine. Our observations of distinct SV2A expression levels by naive CD8^+^ T cells in particular in patients with good versus limited response to LEV led us to validate the relationship between SV2A expression by peripheral immune cells and CNS‐intrinsic cell types in rodents. As a result, we found a significant correlation between the expression of SV2A by NLCs and circulating immune cells, which supports the potential of peripheral SV2A expression as a predictive biomarker for LEV treatment response.

Furthermore, both the consistency of the expression values independent of potential confounding factors such as gender, age, duration of therapy, and LEV dose, and the stable naive CD8^+^‐specific SV2A MFI levels in repeated measures under therapy indicate that SV2A MFI levels in naive CD8^+^ T cells is not subject to dynamic regulation and can thus serve as a robust classifier for predicting LEV treatment response.

### 4.1 Limitations

Our study suffered from several limitations. The study cohort was recruited in a single‐center design with limited group sizes; therefore, it appears recommendable that these results be replicated in a larger cohort with a multicenter approach to increase patient numbers and improve the quality of discrimination of RESs and NRs using the SV2A MFI ratio in CD8^+^ T cells. Next, the clinical response to LEV was measured using relative changes of seizure frequency and discriminating between <50%, >50%, 75%, and 100%. However, these numbers were obtained from the clinical documentation and not from patients' seizure diaries and are therefore susceptible to subjective perception, which in the light of unaware focal seizures can only result in a semiquantitative estimate and does not allow for a more detailed analysis of treatment response (e.g., partial effects on different types of seizures or epilepsy syndromes). Also, a PPV of 82% translates to a sufficiently good sensitivity; however, for a commonly and readily administered drug, the benefit is somewhat limited, and a higher specificity than an NPV of 52% would be more helpful in some clinical decisions. Although we provide statistically significant group differences (*p* = .0010) with promising PPV/NPV values, individual‐level overlap of MFI values between groups indicates that the SV2A MFI ratio represents an innovative exploratory biomarker that warrants prospective validation in independent cohorts to establish its potential for clinical application. Finally, the argumentative connection of SV2A expression in the CNS and the circulating immune cells is based on the correlation between these cell types in a rodent model and not in humans; this is a viable alternative in the absence of access to sufficient amounts of brain tissue but cannot be transferred to humans with absolute certainty or to a clear extent.

## 5 CONCLUSIONS

In conclusion, these data suggest that normalized, naive CD8+ T cell‐specific SV2A MFI levels could potentially serve as a predictive marker for LEV treatment response, significantly enhancing the clinical decision‐making processes. The naive CD8+ T cell‐specific SV2A MFI ratio discriminates well between PWE with good versus poor treatment responses to LEV. Due to the correlation of SV2A expression on circulating and resident CNS cells, the stable expression under continuous LEV treatment, and the relative ease of obtaining them from peripheral blood samples, they may serve as a predictive marker of treatment response to guide clinical decision‐making. Further prospective multicenter studies are recommended to demonstrate robustness and refine the diagnostic value in clinical routine.

## AUTHOR CONTRIBUTIONS

*Conceptualization:* Hajo Hamer and Johannes Lang. *Methodology:* Johannes Lang, Veit Rothhammer, and Hajo Hamer. *Investigation:* Johannes Lang and Jeanne Cuny (blood samples, participant recruitment); Mathias Linnerbauer (animal samples). *Formal analysis:* Johannes Lang and M. Linnerbauer. *Visualization:* Johannes Lang and Mathias Linnerbauer. *Writing—original draft:* Johannes Lang and Mathias Linnerbauer. *Writing—review & editing:* Jeanne Cuny, Veit Rothhammer, and Hajo Hamer. *Experimental optimization (animals & cells):* Veit Rothhammer and Mathias Linnerbauer. *Supervision:* Hajo Hamer.

## CONFLICT OF INTEREST STATEMENT

J.L. has served on the speakers' bureau of UCB Pharma and Destin, outside the submitted work. H.H. has served on the scientific advisory board of UCB Pharma. He has served on the speakers' bureau of or received unrestricted grants from Desitin, Pfizer, and UCB Pharma, outside the submitted work, which manufacture levetiracetam among other antiseizure drugs. V.R. was supported by a Heisenberg fellowship and material resources support provided by the German Research Foundation (RO4866‐3/1, RO4866‐4/1) as well as in transregional and collaborative research centers provided by the German Research Foundation (Project ID 408885537—TRR 274, Project ID 261193037—CRC 1181, Project ID 270949263—GRK 2162, Project ID 505539112—GB.com). M.L. and J.C. have no conflicts of interest. We confirm that we have read the Journal's position on issues involved in ethical publication and affirm that this report is consistent with those guidelines.

## Supporting information


Data S1.

## Data Availability

The data underlying this article cannot be shared publicly, because the public sharing of the data was not included in the written consent information. However, the data will be shared upon reasonable request to the corresponding author.
