# Supplementary material for: SV2A expression in blood cells as a possible biomarker candidate for levetiracetam treatment response
Source: Epilepsia. 2026 Feb 4;67(5):2601–12. doi: 10.1002/epi.70122 (PMC13179651; doi:10.1002/epi.70122)
Supplement: Supplementary file 1 — Data S1. [file EPI-67-2601-s001.docx]

Supplementary material

# Supplementary figures


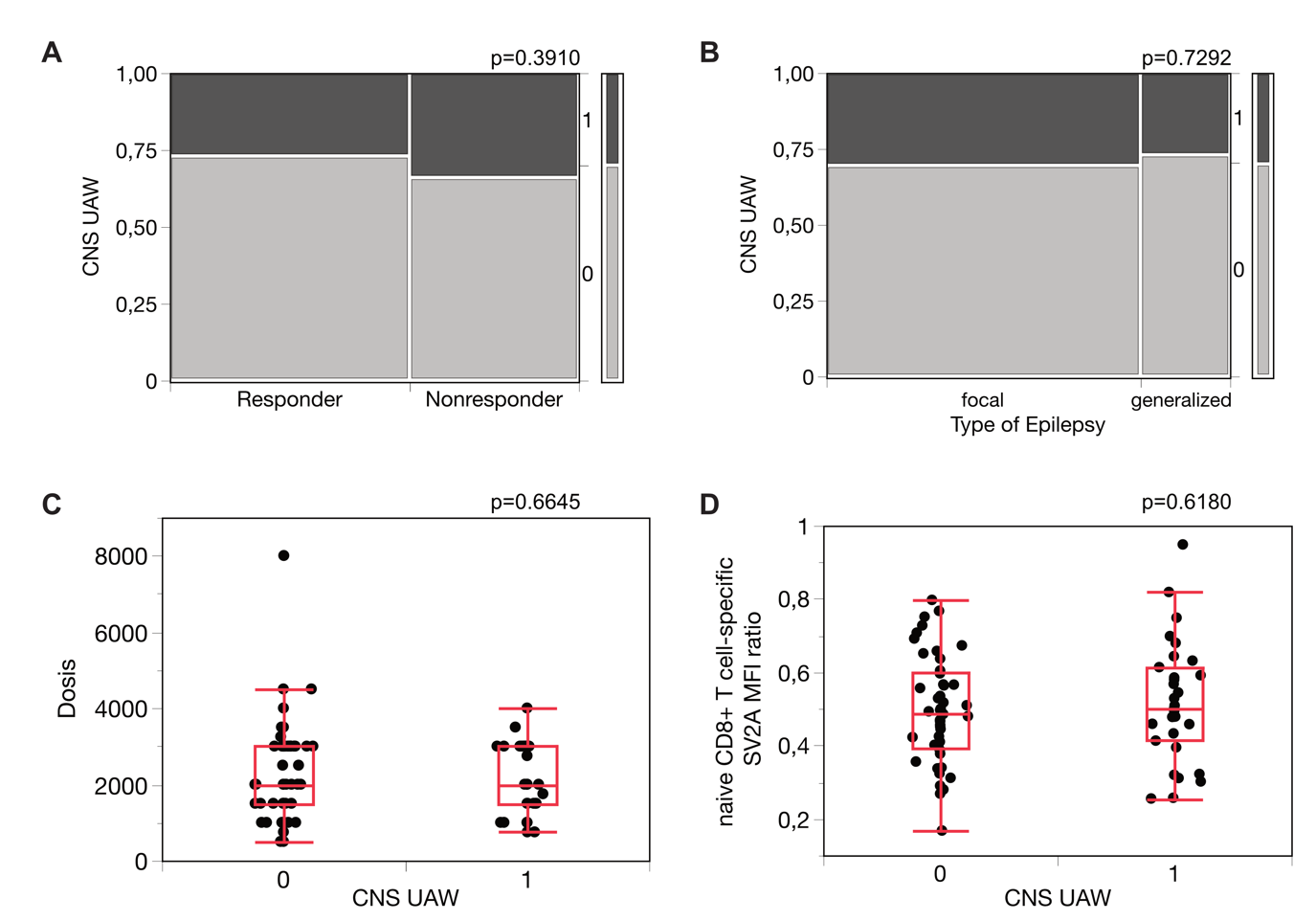


Supplementary Fig 1: CNS UAW. No significant difference when comparing A) Responder vs. Nonresponder (p=0.3910), B) Type of epilepsy (p=0.7292), C) LEV dosis (in mg) (p=0.6645), and D) naïve CD8+ T cell-specific SV2A MFI ratio.


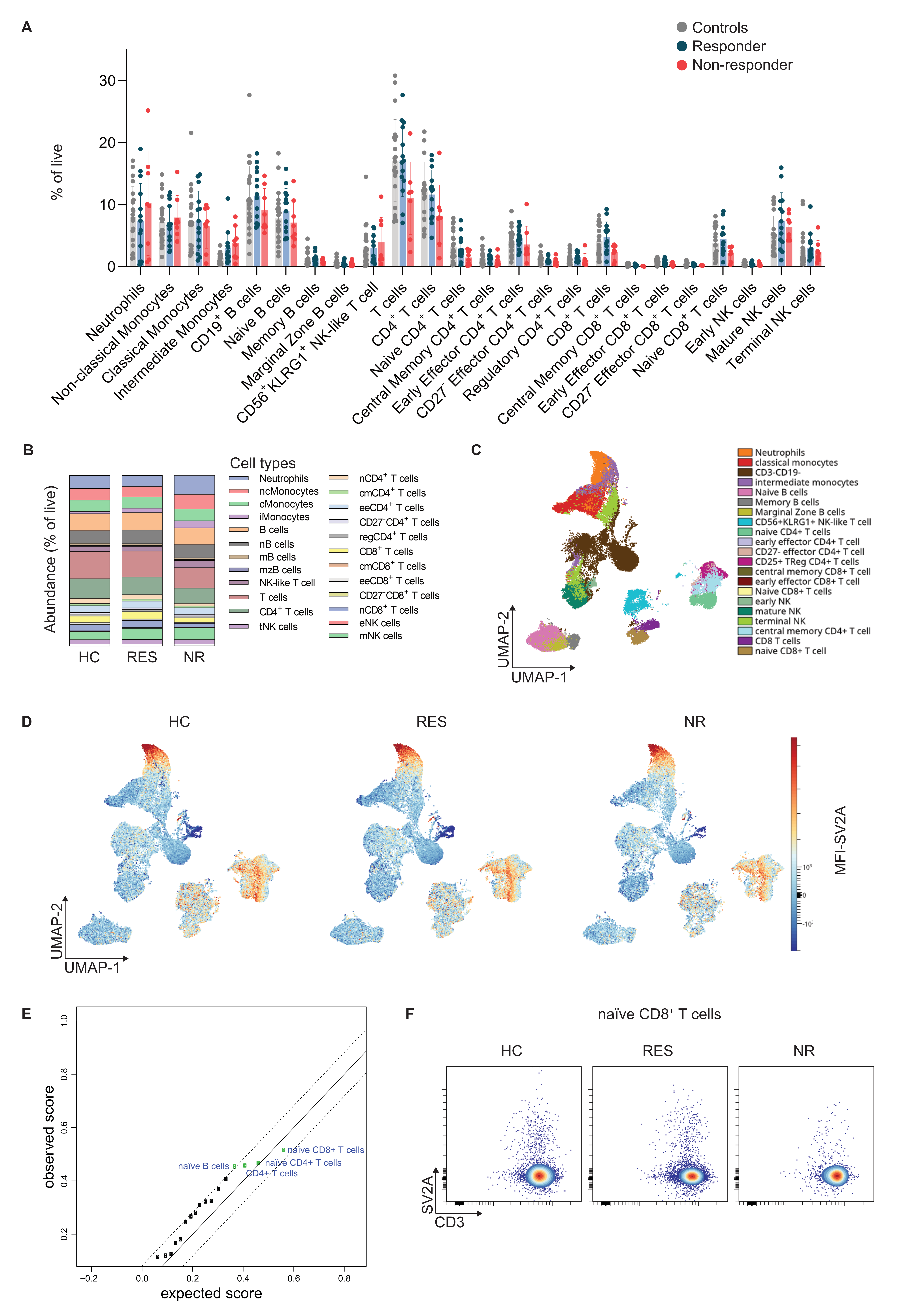


Supplementary Fig 2:

**A.** Abundance (% of live cells) of immune cell populations identified by high-dimensional flow cytometry in controls (n = 27), Responder (n = 34), and Non-responder (n = 18) patients. A detailed gating strategy is provided in the Methods section.

**B.** Abundance (% of live cells) of immune cell populations identified by high-dimensional flow cytometry sorted by patient groups.

**C.** Uniform Manifold Approximation and Projection (UMAP) of immune cells in controls, Responder, and Non-responder patients, overlaid with the gating information obtained by manual gating.

**D.** Uniform Manifold Approximation and Projection (UMAP) of immune cells in controls, responder, and non-responder patients. Color indicates the Median Fluorescence Intensity (MFI) of SV2A in the respective cell clusters with high expression of the protein by neutrophils and T cells (for reference of cell types see panel C).

**E.** SAM plot of significant differences in SV2A expression by Naïve CD8^+^ T cells, naïve CD4^+^ T cells, CD4^+^ T cells, and Naïve B cells in controls (n = 27), Responder (n = 34), and Non-responder (n = 18) patients.

**F.** Representative scatter plots of SV2A expression by Naïve CD8^+^ T cells in controls, responder, and non-responder patients.


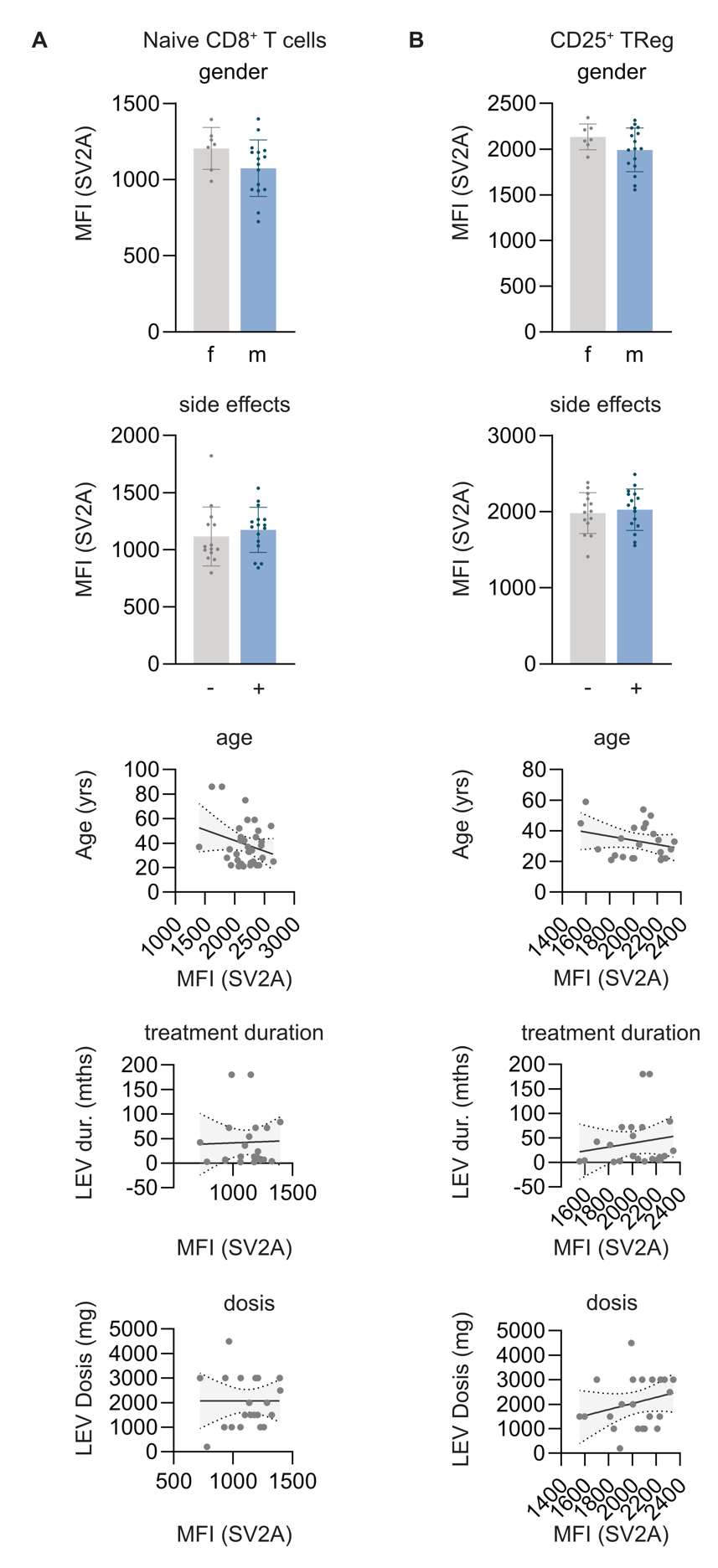


Supplementary Fig 3:

**A.** Confounder Analysis between SV2A levels (MFI) in Naïve CD8^+^ T cells and sex, nervous system side effects, age, Levetiracetam (LEV) treatment duration (in months), LEV dosis (in mg).

**B.** Confounder Analysis between SV2A levels (MFI) in CD25^+^ T_Reg_ cells and sex, nervous system side effects, age, Levetiracetam (LEV) treatment duration (in months), LEV dosis (in mg).

**
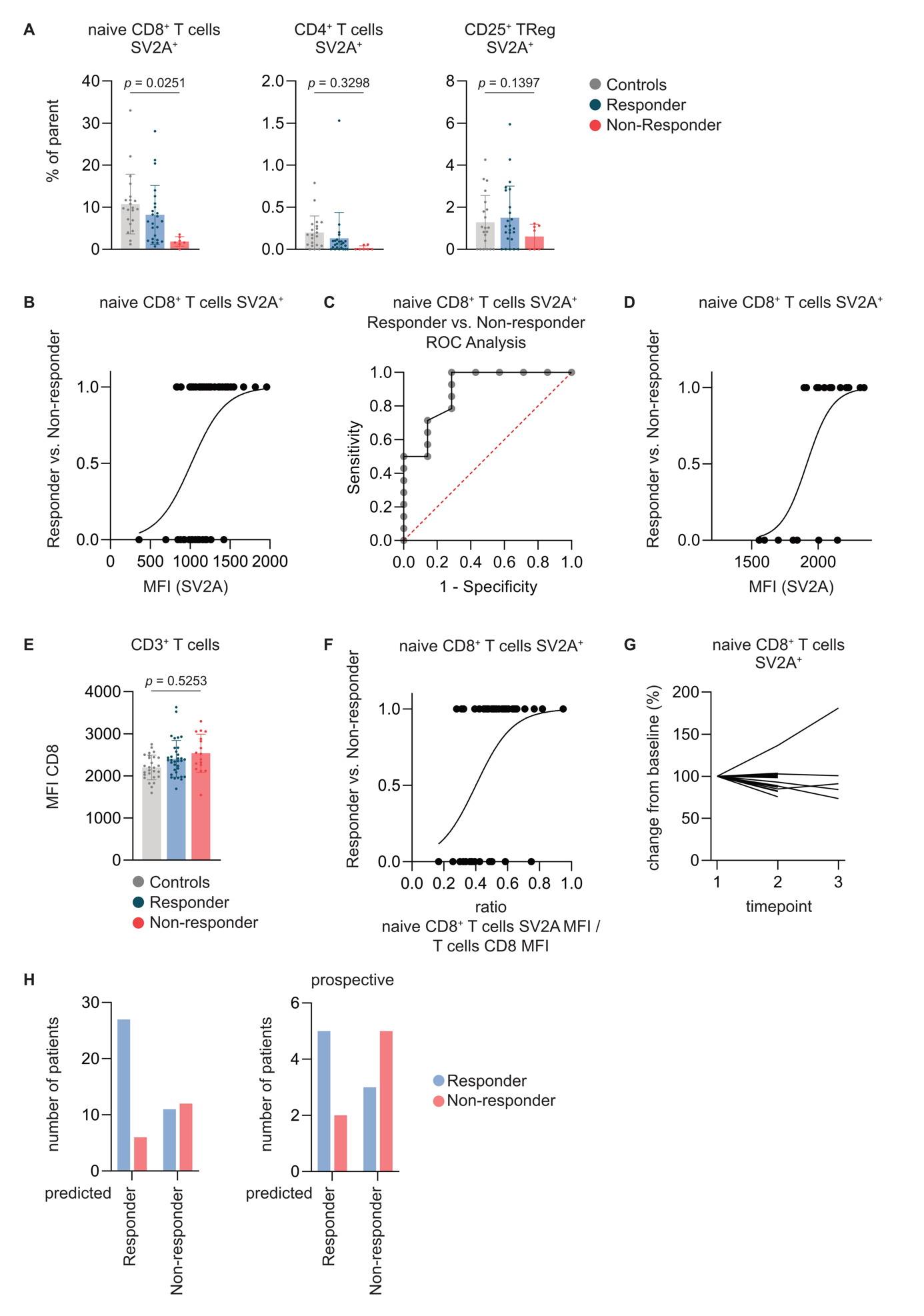
**

Supplementary Fig 4:

**A.** Frequency (% of parent) of SV2A^+^ cells of naïve CD8^+^ T cells, CD4^+^ T cells, or CD25^+^ T_Reg_ cells in control (n = 27), Levetiracetam (LEV)-Responder (n = 34), and LEV-Non-responder (n = 18) patients. Unpaired t-test between Responder and Non-responder.

**B.** Linear regression analysis of SV2A expression (MFI) by Naïve CD8^+^ T cells as classifier for Levetiracetam Responder vs. Non-responder.

**C.** Receiver Operating Characteristic (ROC) curve describing SV2A expression (MFI) by naïve CD8^+^ T cells as classifier for Levetiracetam Responder vs. Non-responder.

**D.** Linear regression analysis of SV2A expression (MFI) by Naïve CD8^+^ T cells as classifier for Levetiracetam Responder vs. Non-responder.

**E.** Longitudinal assessment of Naïve CD8^+^ T cell SV2A levels (MFI) as relative change from baseline in 18 patients with at least two sampling timepoints.

**F.** Median Fluorescence Intensity (MFI) of CD8 expression by CD3^+^ T cells in controls (n = 27), Responder (n = 34), and Non-responder (n = 18) patients. Unpaired t-test between Responder and Non-responder.

**G.** Linear regression analysis of Naïve CD8^+^ T cell SV2A MFI/CD3^+^ T cell CD8 MFI ratio as classifier for Levetiracetam Responder vs. Non-responder.

**H.** Predicted vs. observed response to Levetiracetam. Predicted response was defined as naïve CD8^+^ T cell SV2A MFI/CD3^+^ T cell CD8 MFI ratio above or below the threshold for a retrospective and prospective cohort, p=0.0100 and p=0.3147, respectively (Fisher’s exact test, two sided).


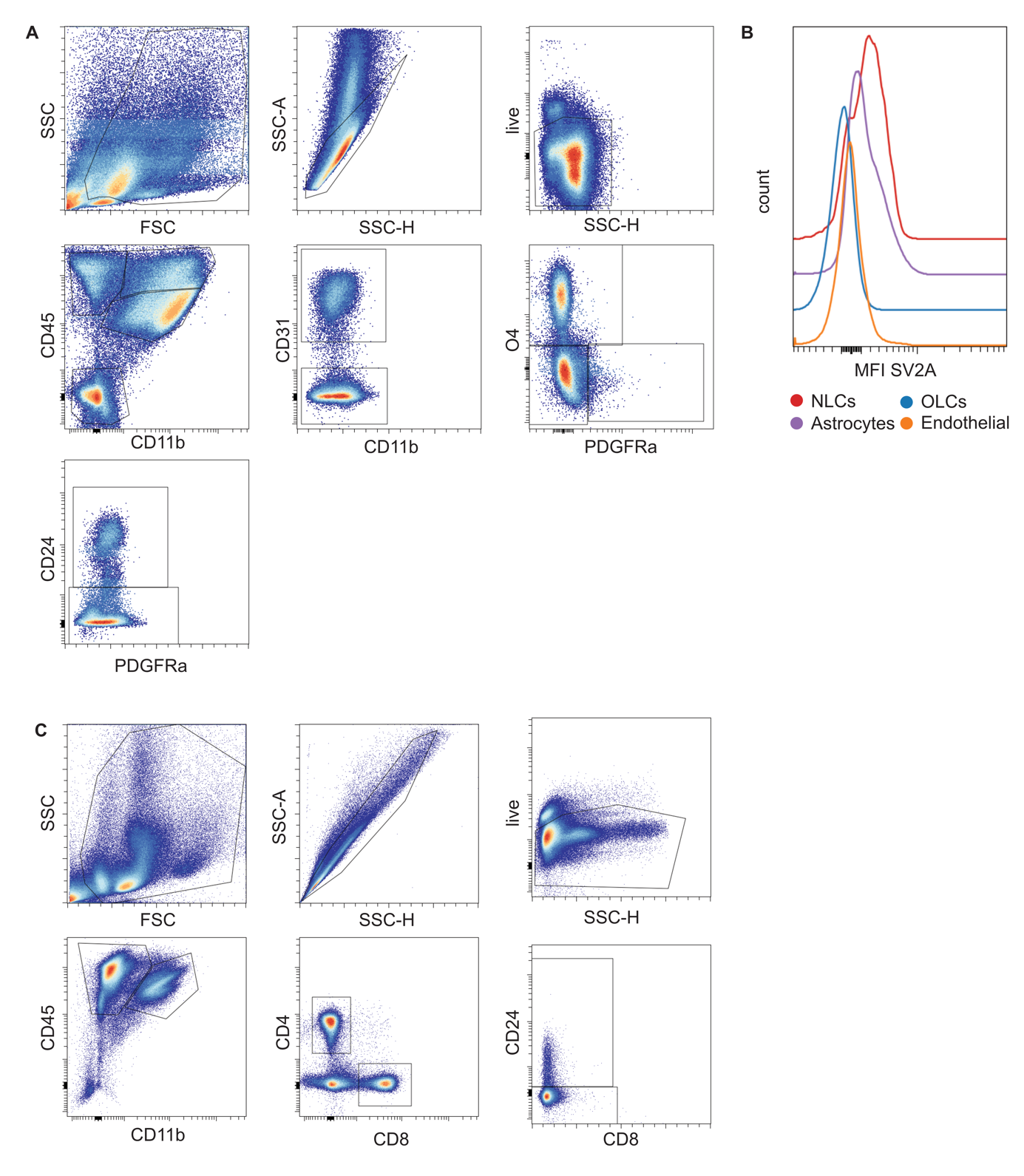


Supplementary Fig 5:

**A.** Gating strategy used for the high-dimensional flow cytometric analysis of SV2A expression by central nervous system (CNS)-cells.

**B.** Histograms depicting SV2A expression by neuronal lineage cells (NLCs), oligodendrocyte lineage cells (OLCs), Astrocytes, and Endothelial cells in wildtype (WT) mice.

**C.** Gating strategy used for the high-dimensional flow cytometric analysis of SV2A expression by splenic and circulating (blood) immune cells.

# Antibodies used in this study

Human:

BV421-CD25 (Thermo Fisher Scientific, # 404025942, 1:100); eFluor 450-CD3 (Thermo Fisher Scientific, #48003742, 1:100); Super Bright 600-CD8 (Thermo Fisher Scientific, #63008842, 1:100); BV650-CD19 (Thermo Fisher Scientific, #416019942, 1:100); BV711-CD56 (Biolegend, #362542, 1:100); Super Bright 780-CD27 (Thermo Fisher Scientific, #78-0279-42, 1:100); FITC-SV2a (Santa Cruz, #sc-376234 FITC, 1:50); PE-KLRG1 (Thermo Fisher Scientific, #12948842, 1:100); PE-eFluor 610-CCR7 (Thermo Fisher Scientific, #61197942, 1:100); PE-Cy5-CD235ab (Biolegend, #306606, 1:200), PE-Fire 700-CD45RA (Biolegend, #304172, 1:200); PerCP-eFluor 710-CD14 (Thermo Fisher Scientific, #46014942, 1:100); PE-Cy7-IgD (Biolegend, #348210, 1:100); Alexa Fluor 700-CD127 (Thermo Fisher Scientific, #56127182, 1:100), APC-eFluor 780-CD16 (Thermo Fisher Scientific, #47016842, 1:100); APC-Fire 810-CD4 (Biolegend, # 344661, 1:100).

Mouse:

BV421-CD11b (Biolegend, #101235; 1:200), BV480-CD11c (BD, #565627, 1:100), BV510-F4/80 (Biolegend,# 123135, 1:100), BV570-Ly6C (Biolegend,# 128029, 1:200), BV605-CD80 (BD, #563052, 1:100), BV650-CD56 (BD, #748098, 1:100), BV650-CD8 (BD, #100741, 1:100), PE-eFlour610-CD140a (Thermo Fisher Scientific, #61140180, 1:100), SuperBright780-MHCII (Thermo Fisher Scientific, #78532080, 1:200), BV711-CD74 (BD, #740748, 1:200), PE-CD45R/B220 (BD, #561878, 1:100), PE-CD105 (Thermo Fisher Scientific, # 12-1051-82, 1:100), PE-Ly6G (BioLegend, #127607, 1:200), PE-CD140a (BioLegend, #135905, 1:100), PE-O4 (Miltenyi, # 130117507, 1:100), PE-Ter119 (Biolegend, #116207), PE-Ly6C (Biolegend, #128007, 1:100), AF488-A2B5 (Novus Biologicals, #FAB1416G, 1:100), PE-Cy5-CD24 (Biolegend, #101811, 1:200), PE-Cy7-CD31 (Thermo Fisher Scientific, #25031182, 1:200), PerCP-eFlour710-CD86 (Thermo Fisher Scientific, #46086280, 1:100), AF532-CD44 (Thermo Fisher Scientific, #58044182, 1:100), PE-Cy5.5-CD45 (Thermo Fisher Scientific, #35045180, 1:300), JF646-MBP (Novus Biologicals, #NBP2-22121JF646, 1:100), APC-Cy7-HB-EGF (Bioss, #BS-3576R-APC-CY7, 1:100), APC-Cy7-Ly6G (Biolegend, #127623, 1:200), AF700-O4 (R&D, #FAB1326N, 1:200, 1:100), BUV737-CD154 (BD, #741735, 1:100), AF660-CD19 (Thermo Fisher Scientific, #606019380, 1:100), APC/Fire810-CD4 (Biolegend, #100479, 1:100).
